# Concerns in the Norwegian Population during the Initial Lockdown Due to the COVID-19 Pandemic

**DOI:** 10.3390/ijerph18116119

**Published:** 2021-06-06

**Authors:** Inger Schou-Bredal, Laila Skogstad, Tine K. Grimholt, Tore Bonsaksen, Øivind Ekeberg, Trond Heir

**Affiliations:** 1 Faculty of Medicine, Institute for Health and Science, University in Oslo, 0130 Oslo, Norway; 2 Department of Research, Sunnaas Rehabilitation Hospital HF, 1453 Bjørnemyr, Norway; uxlask@sunnaas.no; 3 Department of Nursing and Health Promotion, Faculty of Health Sciences, Metropolitan University, 0130 Oslo, Norway; 4 Faculty of Health Studies, VID Specialized University, 0319 Oslo, Norway; tine.grimholt@vid.no; 5 Department of Acute Medicine, University Hospital, 0424 Oslo, Norway; 6 Department of Health and Nursing Sciences, Faculty of Social and Health Sciences, Inland Norway University of Applied Sciences, 2418 Elverum, Norway; tore.bonsaksen@inn.no; 7 Faculty of Health Studies, VID Specialized University, 4306 Sandnes, Norway; 8 Psychosomatic and CI Psychiatry, Division of Mental Health and Addiction, Oslo University Hospital, 0424 Oslo, Norway; oeekeber@online.no; 9 Norwegian Center for Violence and Traumatic Stress Studies, 0409 Oslo, Norway; trond.heir@medisin.uio.no; 10 Institute of Clinical Medicine, University of Oslo, 0316 Oslo, Norway

**Keywords:** concern, COVID-19, lockdown, population study, survey, worries

## Abstract

Although concern affects one’s welfare or happiness, few studies to date have focused on peoples’ concerns during the initial COVID-19 lockdown. The aim of the study was to explore concerns in the Norwegian populations according to gender and age, and identify which concerns were most prominent during the lockdown. A population-based cross-sectional online survey using snowball-sampling strategies was conducted, to which 4527 adults (≥18 years) responded. Questions related to concerns had response alternatives yes or no. In addition, they were asked which concern was most prominent. Nearly all the 4527 respondents (92%) reported that they were concerned: 60.9% were generally concerned about the pandemic, 83.9% were concerned about family and friends, 21.8% had financial concerns, and 25.3% expected financial loss. More women were concerned about family and friends than males, (85.2% vs. 76.2%, *p* < 0.001), whereas more men expected financial loss (30.4% vs. 24.4%y, *p* = 0.001). Younger adults (<50 years) had more financial concerns than older adults (25.9% vs. 10.5%, *p* < 0.001). Being concerned about family and friends was the most prominent concern and was associated with; lower age (OR 0.79), female gender (OR 1.59), and being next of kin (2.42). The most prominent concern for adults 70 years or older was being infected by COVID-19. In conclusion, women and younger individuals were most concerned. While adults under 70 years of age were most concerned about family and friends and adults 70 years or older were most concerned about being infected by COVID-19.

## 1. Introduction

In Norway, the lockdown due to the Coronavirus Disease 2019 (COVID-19) outbreak was initiated in mid-March 2020 and gradually increased in magnitude. People were advised to reduce face-to-face interaction and isolate themselves at home. All schools, kindergartens, and universities were closed, and schooling was continued (partially) through digital platforms such as zoom or teams. Many of the working adults were either instructed to work from home, temporarily laid off, or put on no-pay leave. Traveling restrictions were implemented and ports and airports were closed. Most shops, restaurants, cinemas, and public places were shut down to contain the expansion of COVID-19 and all non-emergency medical treatments were ceased/put on hold.

Several studies have described the impact of the COVID-19 pandemic on people’s psychological well-being. Feelings of separation, apprehension, stress, anxiety, and depression were reported [1,2,3,4,5,6]. The psychological impact of quarantine had substantial negative, psychological impacts on people, contributing to post-traumatic stress symptoms [7,8]. Uncertainty regarding the length of the situation and its final consequences adds to the anguish.

Although it is a part of human nature to worry, during a pandemic, worries are most likely amplified and many of the issues one is concerned about is probably out of ones’ control. In addition, the media usually report on COVID-19 negatively; for example, by reporting the number of people infected and dead giving rise to more concern.

Being worried/concerned affects one’s welfare or happiness. Thus, in addition to collecting information regarding peoples’ mental health during a lockdown due to a pandemic, it is also important to collect information regarding peoples’ worries/concerns. Such information might assist authorities in providing informed interventions and clear communications that may reduce some of the concerns people have.

However, few studies were identified that assessed concern among the public during lockdown. It appears that males tend to report less concern due to COVID-19 than females [9]. In the Italian population, Germani et al., 2020 found that young adults showed more concern for being asymptomatic carriers than being positive with COVID-19 themselves [10]. A study from the United States reported that the top concern of the public was getting sick because of COVID-19 and not being able to obtain medical help [11]. A survey conducted in seven European countries: Denmark, France, Germany, Italy, Portugal, the Netherlands, and the UK, found that people in Portugal and Italy were most concerned with the economic consequences of the pandemic [12]. A survey conducted in the United States, United Kingdom, and Germany found that the main concern among the population was for their family’s health, followed by concerns regarding their country’s economic stability.

### Aims

To explore concerns (i.e., contagion, financial, family and friends) in the Norwegian populations according to gender and age, and which concerns were most prominent during lockdown.

## 2. Materials and Methods

### 2.1. Design

A population-based cross-sectional survey (CORONAPOP) was conducted between 8 April 2020 and 20 May 2020. Data were collected by means of an open web-link, which was disseminated from several institutions, including Oslo University Hospital, Sunnaas Rehabilitation Hospital, and the University of Oslo. In addition, the web link was disseminated on social media platforms such as Facebook, Twitter, LinkedIn, Instagram, and was also featured in national and local newspapers with online links.

### 2.2. Sample

The study participants were Norwegian citizens aged 18 years or older. There were no exclusion criteria.

### 2.3. Measures

All data collected was self-reported. The survey employed several measures identical to the ones used in the Norwegian Population (NORPOP) health survey, which was conducted as a postal survey from 2014 to 2015 [13,14,15].

### 2.4. Sociodemographic Variables

Sociodemographic data collected were: age, gender, cohabitation status (living with spouse or partner versus not), living with parents, living with children <18 years of age, education level (lower versus higher education defined as more than four years of higher education (i.e., College, University), employment status before and during the COVID-19 outbreak and social support. Employment was dichotomized, working/paid leave/in education versus not and size of workplace (<200 inhabitants (rural), 200–19,999 inhabitants, 20,000–99,999 inhabitants, 100,000 inhabitants or more (urban). Social support was assessed with the following question: “Do you have friends that will provide help when you need it?” Response options were yes or no.

### 2.5. Concerns Related to COVID-19

A questionnaire was developed to assess concerns related to the COVID-19 pandemic. The participants were asked to indicate (yes or no) whether they had general concerns in relation to COVID-19, concerns for family and friends, financial concerns, and/or expected financial loss in the future. In addition, the participants were asked which one of the listed items was currently most discomforting. The listed items were: financial concern, expecting financial loss in the future, being infected by COVID-19, quarantine or isolation, being at risk of complications if infected by COVID-19, concern for family and friends, or being generally concerned about the pandemic. We also included an open-ended question: “Do you have other concerns related to the pandemic? If so, please elaborate on your concerns”.

### 2.6. Problems Related to COVID-19

The participants were asked if they had been infected with coronavirus, had been quarantined or in isolation, or defined themselves as being at risk for complications if infected by coronavirus. In addition, they were asked if they, during the initial phase of the lockdown, had been a patient or next of kin. The response options were yes or no.

### 2.7. Statistical Analyses

Participants who answered yes to being generally concerned, concerned for family and friends, or reported financial concerns were classified as being concerned. Characteristics of those who were concerned and those who were not are presented separately as frequencies and percentages. Between-group comparisons were conducted using the chi-squared test in the case of categorical variables. We conducted post hoc analyses such as unadjusted and adjusted logistic regression to examine the associations between sociodemographic variables and variables related to COVID-19 (independent variables) and being concerned for family and friends as the dependent variable. To reduce the type 1 error probability a lower significance level was chosen. All tests were two-tailed, and differences were considered significant if *p* < 0.01. Windows IBM SPSS Statistics (version 24, IBM Corp., Armonk, NY, USA) was used for statistical analyses.

### 2.8. Ethics

Ethical approval for conducting the study was granted from the Regional Committee for Medical and Healthcare Ethics (REK no. 130447). The principles in the Declaration of Helsinki were respected. The questionnaires were completed anonymously. Contact information was provided as a possibility to mail the research team in case of health care/psychological support needs.

## 3. Results

### 3.1. Survey Respondents

A total of 4,527 individuals responded to the online questionnaire. The majority were women (85.0%), had higher education (75.5%), were under 60 years of age (88.3%), and lived in cities (46.3%). Only a few (1.4%) had been infected by COVID-19, 28.2% had been in quarantine or isolation and 23.4% reported having risk factors for developing complications if infected by COVID-19 (Table 1). Nearly all (90.5%) reported that they had someone that would help if needed. During the outbreak, 2.9% of the individuals had been hospitalized due to COVID-19 or other diseases, of these, 18.9% had been infected by COVID-19. Of the 271 (6.0%) who had been next of kin during the lockdown, 90% were women. Of those who had been in quarantine or isolation, 4.5% had been infected by COVID-19.

### 3.2. Concerns

The majority (91.9%) reported that they were concerned. A total of 60.7% were generally concerned due to the pandemic, 83.9% were concerned for family and friends, 21.8% had current financial concerns and 25.3% expected financial loss in the future.

### 3.3. Concerns According to Gender

More women than men reported that they had friends who would provide them with support if needed, 91.1% vs. 86.8%, *p* < 0.001. More men than women reported to be at risk of complications if they were infected with coronavirus, 29.8% vs. 22.4%, *p* < 0.001. There was no difference between the gender with regard to being concerned about getting infected by COVID-19, 12.3% men vs. 12.3% women, *p* = 0.42.

More women than men reported that they were concerned, respectively, 92.6% vs. 88.2%, *p* < 0.001. Women were more likely to be concerned about family and friends than men, 85.2% vs. 76.2%, *p* < 0.0001, whereas men were more likely to expect financial loss in the future due to the pandemic (30.4% vs. 24.4%, *p* = 0.001). There was no difference between the genders with regard to having financial concern, women 21.9% vs. men 20.9%, *p* = 0.61.

### 3.4. Concerns According to Age

Significantly more younger adults (<50 years of age) reported that they were concerned compared to the elder (>50 years of age), 93.1% vs. 88.7%, *p* < 0.001. Young adults had more financial concerns compared to the elder, 25.9% vs. 10.5%, *p* ≤ 0.001 and were more inclined to expect financial loss in the future, 27.1% vs. 20.3%, *p* < 0.001.

### 3.5. Most Prominent Concerns during Lockdown

As shown in Table 2, being concerned for family and friends (38.1%) was the most prominent concern during the lockdown, followed by being generally concerned about the COVID-19 pandemic (14.7%) and being infected by COVID-19 (13.4%). For a small percentage (6.8%) their most prominent concern was not listed, but they had been given the opportunity to write these down. Examples of what they were concerned about were: the consequences for society and the duration of the pandemic just to mention a few [16]. 

### 3.6. Factors Associated with Being Concerned for Family and Friends

The associations between the independent variables and being concerned for family and friends are shown in Table 3. The risk for being concerned for family and friends was higher for individuals under 50 years of age, women, having been next of kin, and having been in quarantine or isolation. The multiple logistic regression analysis including all independent variables showed that the risk of being concerned for family and friends was higher among women (OR 1.52, *p* < 0.001), those who were married or cohabiting (OR 1.12, *p* < 0.001), those who had been next of kin (OR 2.42, *p* < 0.001), and among those who were under 50 years of age (OR 0.79, *p* < 001).

## 4. Discussion

The majority of the population was concerned due to the COVID-19 pandemic. However, women had more COVID-19 related concerns than men. Individuals under 50 years of age more often had financial concerns and expected financial loss compared to individuals 50 years or older. The majority were concerned for family, friends, and being infected. For individuals 69 years or younger, the most prominent concern during the initial lockdown was for family and friends. For individuals 70 years or older, the most prominent concern was being infected by COVID-19.

### 4.1. Gender and Age Groups

Our finding that more women were concerned due to the COVID-19 pandemic compared to men is similar to previous studies [7,9,17]. One possible explanation is that women carry a disproportionately greater share of the COVID-19-related care-giving load at home, such as childcare and remote schooling [18,19,20]. Another explanation could be that women provide the majority of informal care to spouses, parents, parents-in-law, friends, and neighbors [21].

At the beginning of the pandemic, experts and social media emphasized that younger individuals were not affected or mildly affected by the COVID-19 virus, and it was considered similar to the common flu and a disease of the elderly [22]. This is probably the reason why younger adults were less concerned about being infected by the COVID-19 then, but overall, younger adults in the present study were more concerned during the lockdown. There could be several reasons for this. According to a report by the International Labor Organization (ILO), more than 70% of youth who study or combine study with work were adversely affected by the closing of schools, universities, and training centers since the outset of the pandemic [23]. According to their report, 65% of the people reported having learned less since the outbreak of the pandemic because of the transition from classroom to online and distance learning. This may create uncertainty as to whether they will be able to complete their education, which in turn can create uncertainty regarding getting a job in the future. Furthermore, students often have part-time jobs in shops and restaurants/pubs, which were closed leading to less income and thus, concerns about the economy. Young adults are also considered the key active working force in society and are therefore more strongly affected by business closure and redundancies. Thus, young adults could be more concerned regarding future consequences and economic challenges caused by the pandemic than elders could. Our finding that more adults under 50 years of age expected financial loss in the future supports this view.

### 4.2. Being Concerned for Family and Friends Was Most Prominent during Lockdown

Risk perception and worries about the contagion are psychological aspects that are particularly relevant in a pandemic scenario, as they shape social reactions and behavior changes [24]. However, in the present study, this was not the most prominent concern. Being concerned for family and friends was the most prominent concern during lockdown for both genders. People were concerned that someone in their family or close friends would be infected by COVID-19 or what the isolation would do to their mental health. Many elders were isolated, without physical contact with their children or grandchildren. Others had their family or children abroad and could not visit. Our finding is similar to the opinion survey Norwegian Corona monitor (The Norwegian Corona Monitor survey) which has mapped the social consequences of COVID-19 since 13 March [25]. According to that survey, people are more concerned about others than about themselves. Nine out of 10 avoided socializing with family and friends because of COVID-19. Additionally, in the United Kingdom, United States, Germany and China the main concern was for their family’s health [16,26]. According to Barzilay et al. [17] being most concerned about family and friends during the pandemic may be related to “tend-and-befriend”, where in response to threat, humans tend to protect their close ones (tending) and seek out their social group for mutual defense (befriending) [27]. Thus, the findings that people are more concerned about others than themselves during the pandemic outbreak might, according to Barzilay et al., (2020) be interpreted as a form of altruism.

Being female, young, and next of kin were the strongest associations with being concerned for family and friends. This is not surprising since, as mentioned earlier, women provide the majority of informal care to spouses, parents, parents-in-law, friends, and neighbors [20]. It seems feasible that being next of kin to a family member or friend would amplify one’s worry for that person during the pandemic. One would naturally be worried that the patient would contract the virus or if they as next of kin are unknowingly spreading the virus to them. However, when analyzing age groups, we found that the older adults (70 years or older) most prominent concern was being infected by COVID-19. This was not surprising since it had been emphasized in the media that older adults are at a significantly higher risk of severe disease following infection from COVID-19.

### 4.3. Study Limitations and Strengths

We presented data from a large sample in the initial phase of the COVID-19 lockdown with severe social distancing. However, some limitations should be addressed. The link to the survey was distributed openly on the internet via social media channels, while this is an effective recruitment strategy, the self-selected sample was not representative of the general population in terms of age, gender, and education. The responders were mainly young, women, well educated, and urban. We used binary (yes/no) answers in measures in the study, which are insensitive, and likely to miss some important nuances. However, by using the same scale, comparisons can be made. Furthermore, the focus was if individuals were concerned, and not how much they were concerned. The cross-sectional design does not allow causal inferences, but this could be addressed in future longitudinal studies. We did not correct for the possibility of type 1 errors when performing multiple hypotheses tests. Thus, our results may be vulnerable to false discoveries. However, to reduce the type 1 error probability, a lower significance level, *p* = 0.01 was chosen.

## 5. Conclusions

The majority of the population reported that they were concerned in the initial phase and in lockdown due to the COVID-19 pandemic. Women and younger individuals were more concerned during the initial lockdown compared to their counterparts. While adults below 70 years were most concerned about family and friends, adults 70 years or older were more concerned about being infected by COVID-19. This information might assist authorities in providing informed interventions and clear communications that may reduce some of the concerns people have.

## Figures and Tables

**Table 1:** Sample characteristics (*n* = 4527).

Variables	Generally Concerned	Not Concerned	*p*
	(*n* = 4162)	(*n* = 365)	
	%(*n*)	%(*n*)	
Age group			<0.0001
<30	26.5 (1101)	15.1 (55)	
30–39	27.2 (1130)	24.7 (90)	
40–49	20.4 (849)	22.5 (82)	
50–59	16.4 (684)	22.5 (82)	
60–69	7.5 (311)	11.8 (43)	
70–79	1.8 (73)	2.5 (9)	
80 years or more	0.3 (14)	1.1 (4)	
Gender			<0.0001
Female	86.0 (3565)	78.5 (285)	
Male	14.0 (581)	21.5 (78)	
Higher education	75.4 (3140)	75.9 (277)	0.89
Size of place of residence			0.18
<2000 inhabitants	4.0 (167)	5.5 (20)	
2000–19,999 inhabitants	25.5 (1061)	21.9 (80)	
20,000–99,999 inhabitants	23.9 (993)	26.8 (98)	
100,000 inhabitants or more	46.4 (1932)	45.5 (166)	
Married/cohabiting	60.3 (2510)	56.4 (206)	0.16
Living alone	21.5 (894)	27.4 (100)	0.01
Living with parents	7.1 (294)	6.8 (25)	0.99
Living with children <18 years of age	34.3 (1429)	32.3 (118)	0.46
Employment/in education/paid leave			
During outbreak of COVID-19	80.7 (3358)	84.7 (309)	0.06
Before outbreak of COVID-19	88:0 (3663)	85.8 (313)	0.21
Problems related to COVID-19			
COVID-19 infected	1.4 (57)	2.2 (8)	0.24
Been in quarantine or isolation	28.6 (1192)	23.6 (86)	0.04
Risk of complication	23.7 (986)	20.5 (75)	0.20
Was a patient during outbreak	2.9 (121)	3.0 (11)	0.87
Was next of kin during outbreak	6.3 (262)	2.5 (9)	0.002

Note. Statistical tests are Chi-Square tests. Missing data ranged from 0.00% to 0.64%. Higher education is more than four years of College or University.

**Table 2:** Concerns that created the most discomfort.

Concerns.	Total % *(n*)	Gender		Age Group					
		Females	Males	<30	30–39	40–49	50–59	60–69	70 +
		% (*n*)	% (*n*)	% (*n*)	% (*n*)	% (*n*)	% (*n*)	% (*n*)	% (*n*)
Being infected	13.4 (608)	13.6 (522)	12.3 (81)	8.5 (98)	10.4 (109)	15.4 (143)	17.4 (133)	22.0 (78)	29.0 (29)
Financial	11.9 (536)	10.8 (418)	17.6 (116)	11.7 (135)	10.0 (122)	13.7 (128)	11.7 (90)	13.3 (47)	15.0 (15)
Financial loss	2.5 (112)	1.9 (73)	5.9 (39)	2.2 (25)	8.9 (109)	2.6 (24)	2.1 (16)	3.4 (12)	1.0 (1)
Family and friends	38.1 (1724)	38.8 (1495)	34.0 (2249)	45.3 (524)	38.9 (474)	34.0 (317)	38.1 (292)	26.3 (93)	24.0 (24)
General concerns	14.7 (664)	15.4 (591)	10.5 (45)	16.3 (188)	17.2 (210)	13.0 (121)	12.3 (94)	12.1 (43)	8.0 (8)
Other	6.8 (310)	6.0 (264)	6.8 (45)	6.4 (74)	8.0 (97)	7.3 (68)	6.5 (50)	5.6 (20)	1.0 (1)

Note. Financial means currently having concerns regarding one’s own finances. Financial loss is expecting the COVID-19 situation to cause personal financial loss in the future. However, when looking at the age groups separately, the most prominent concern among those who were 70 years or older was being infected by COVID-19.

**Table 3:** Associated factors with being concerned for family and friends.

Independent Variables	Unadjusted			Adjusted		
	OR	95% C.I	*p* Value	OR	95% C.I	*p*
Younger age group (<50 years)	0.81	0.76–0.85	<0.0001	0.79	0.74–0.84	<0.001
Female gender	1.76	1.46–2.18	<0.0001	1.59	1.29–1.95	<0.001
Higher education	0.96	0.79–1.15	0.62	0.93	0.77–1.14	0.49
Married/cohabitate	1.12	0.95–1.31	0.17	1.32	1.11–1.56	0.001
Live in an urban setting	1.34	0.94–1.92	0.11	1.29	0.89–1.86	0.18
Not employed/in education/paid leave	1.13	0.93–1.38	0.21	1.01	0.82–1.26	0.93
Have social support	1.11	0.85–1.44	0.44	1.02	0.78–1.35	0.87
Patient during outbreak	1.08	0.67–1.75	0.75	1.23	0.72–2.08	0.45
Next of kin during outbreak	2.38	1.52–3.75	<0.0001	2.42	1.53–3.81	<0.001
Have been infected by COVID-19	0.69	0.38–1.27	0.24	0.61	0.32–1.17	0.14
Have been in quarantine/isolation	1.31	1.09–1.58	0.004	1.25	1.03–1.52	0.022
Risk of complication	0.97	0.80–1.17	0.73	1.30	1.05–1.61	0.014
Hosmer and Lemeshow Test, 0.23						

Note. Dependent variable being concerned for family and friends due to COVID-19. Social support is having someone that will help if needed. Risk of complications is self-reported risk of complications in the case of contracting the coronavirus. Age groups are in ten-year intervals.

## Data Availability

The datasets used and/or analyzed during the current study are available from the corresponding author on reasonable request.
